# Impact of the Nationwide Avastin Ban at a Tertiary Eye Care Hospital in Pakistan

**DOI:** 10.7759/cureus.65199

**Published:** 2024-07-23

**Authors:** Fiza Shaheen, Huzaifa Farooq, Muhammad Amjad

**Affiliations:** 1 Ophthalmology, Al-Shifa Trust Eye Hospital, Rawalpindi, PAK; 2 Vitreoretina, Al-Shifa Trust Eye Hospital, Rawalpindi, PAK

**Keywords:** sterilization, ranibizumab, intravitreal injections, endophthalmitis, bevacizumab

## Abstract

Objective: To report the impact of a 10-week-long nationwide ban on intra-vitreal bevacizumab (IVB) injection (Avastin®) at a tertiary care hospital in Pakistan.

Methods: This was a single-center, retrospective, cohort study. Patients scheduled for IVB injections from October 25, 2023 to October 29, 2023 who arrived in OPD between November 28, 2023 and December 15, 2023 for their assessment were included in this study.

Results: Among the identified 412 patients, only 103 met the inclusion criteria. The mean age was 59.35 ± 9.5 (mean ± SD). About 60.2% were male (n = 62). Diabetic macular edema (DME) was the most common indication (n = 71, 68.9%). The mean total duration of treatment delay was 81.67 ± 17.15 days. While the delay due to the Avastin® ban was 67.47 ± 4.8 days. Eyes that had not received any prior injections were 46 (44.7%) while others had received at least 1 (n = 43, 41.7%) or 2 injections (n = 14, 13.6%) before. Mean central macular thickness (CMT) before and after treatment delay was 362.7 ± 113.4 μm and 398.38 ± 124 μm (p < 0.05), respectively. Among 20 patients with vitreous hemorrhage (VH), 14 patients showed marked improvement (70%), 5 showed no change in severity (20%) and 1 (5%) had further worsening. CMT difference was strongly correlated with the total duration of treatment delay (p < 0.01) and with the number of injections (p < 0.01).

Conclusion: The nationwide ban on Avastin® heightened the severity of disease in the patients highlighting the delicate balance between safety precautions and timely access to essential medical interventions.

## Introduction

Intravitreal injections (IVIs) are frequently performed in standard ophthalmic practice, with anti-vascular endothelial growth factor (VEGF) agents being the most commonly used. These agents are often employed to treat various conditions characterized by an overexpression of VEGF due to retinal hypoxia, which leads to pathological angiogenesis [1]. However, they also come with potential risks and complications among which infective endophthalmitis (IE) is the most serious one [2] with a reported risk of 0.019% per injection [3].

In our region, three anti-VEGF IVI are accessible: Bevacizumab (Avastin®, Roche, Basel, Switzerland), Ranibizumab (Patizra®, Novartis, Basel, Switzerland), and Aflibercept (EYLEA®, Bayer HealthCare, Berlin, Germany/Regeneron Pharmaceuticals Inc., Tarrytown, USA). Bevacizumab (Avastin®) is the preferred choice due to its cost-effectiveness. These injections have transformed the treatment and prognosis for individuals with age-related macular degeneration (AMD), diabetic macular edema (DME), and retinal vein occlusion (RVO) [4-6].

Bevacizumab (Avastin®), a recombinant humanized monoclonal IgG1 antibody, binds to all isoforms of VEGF. Avastin® was initially approved by the FDA for treating metastatic colorectal cancer [7]; however, it is being used off-label for various eye diseases due to its proven effectiveness for AMD [8]. In Pakistan, the Drug Regulatory Authority of Pakistan (DRAP) issued a suspension of the sale and distribution of Avastin® IVIs on September 25, 2023, following the emergence of at least 68 cases of post-Avastin® IE. Subsequent investigations revealed that the contamination stemmed from a third-party supplier. Despite this clarification, Avastin® was not reintroduced into our setup until December 11, 2023. In our setup, where we typically administer intra-vitreal Avastin® injections (IVB) to at least 80 patients daily, the temporary ban on these injections for approximately 10 weeks had a notable impact on both our services and our patients. Our study seeks to explore how this ban affected our scheduled patients with a range of indications.

## Materials and methods

This was a single-center, retrospective, cohort study that was conducted in the vitreoretina (VR) department of Al-Shifa Trust Eye Hospital (ASTEH), Rawalpindi, Pakistan, after obtaining the Ethical Review Committee (ERC) approval from Al-Shifa Research Center.

A total of 412 patients were identified from their medical record numbers who were scheduled for IVB injections from September 25, 2023, to September 29, 2023. They were called between November 27, 2023 and December 12, 2023 after tracking their mobile numbers from Al-Shifa Electronic Medical Record (EMR). Among the 377 patients who could be contacted, 23 had already received other anti-VEGF IVI (Patizra/Eylea) privately and were therefore excluded from the study. Only 103 patients attended our OPD for evaluation.

Inclusion criteria

All the patients scheduled for IVB injections from October 25, 2023, to October 29, 2023, who arrived in OPD between November 28, 2023 and December 15, 2023 for their assessment were included in this study.

Exclusion criteria

Patients excluded from this study included those who did not attend assessment during the specified dates, those who received other anti-VEGF injections during the Avastin® ban period, and those whose data lacked recent OCT macula information (except for cases of vision-obscuring vitreous hemorrhage (VH)).

In cases of DME, RVO, and wet AMD, we had a pro re nata (PRN) method for managing patients that involved administering an initial set of three IVIs per month, followed by injections as per follow-up appointments. For diabetic VH (DVH), after ruling out underlying traction on B scan ultrasonography, we administered at least one IVB followed by reassessment after six weeks. If there was an improvement but the view was still not sufficient for PRP, one more IVB was given otherwise PRP was done. If there was no improvement at all, then patients were booked for PPV. We attended to a minimum of 80 patients every day for IVB for various conditions. However, due to high patient turnover, those who were advised IVB were scheduled for injections approximately four to six weeks after their assessment.

Patients included in this study had their dilated fundal examination on slit lamp biomicroscopy. Optical coherence tomography (OCT) macula scan (Spectralis OCT (Heidelberg Engineering, Heidelberg, Germany)) was done for the measurement of central macular thickness (CMT) in eyes with DME, RVO and wet AMD or other causes of choroidal neovascularization (CNV). CMT or foveal thickness was defined as the mean thickness within the central 1000-μm diameter area in microns from the OCT macula thickness map (as defined by ETDRS) [9]. The seriousness of each instance of VH was evaluated using a five-point scale established by Lieberman et al. [10]. The scale is as follows: grade 0 (absence of VH); grade 1 (minimal hemorrhage with clear visibility of the optic disk and retinal vessels); grade 2 (mild hemorrhage with most of the optic disk and retinal vessels visible); grade 3 (moderate hemorrhage with the optic disk or retinal vessels barely visible); and grade 4 (severe hemorrhage too dense to allow visualization of the optic disk).

Recorded variables included age, gender, laterality, and diagnosis with the total prior injections if any. The total duration of delay was calculated in the number of days from the advised injection to the day of assessment. While duration of delay due to the Avastin® ban was separately calculated too in the number of days from the scheduled injection to the day of assessment. Before and after the treatment delay CMT was measured with OCT. In cases of VH, severity was compared before and after the treatment delay using the five-point qualitative scale.

Data analysis was done using IBM SPSS Statistics for Windows, Version 21 (Released 2012; IBM Corp., Armonk, New York, United States). For qualitative measures, frequency and percentage were used while mean and standard deviation (mean ± SD) were calculated for quantitative measures. Paired sample t-test was used to compare overall CMT before and after the treatment delay. One sample t-test was used to compare CMT for each diagnosis. Pearson correlation was used to correlate CMT difference with gender, number of previous injections, and duration of treatment delay. A p-value of ≤0.05 was considered statistically significant.

## Results

The mean age of the patients was 59.35 ± 9.5 (mean ± SD). About 60.2% were male (n = 62) with 54.4% right eye involvement (n = 56). DME was the most common indication (n = 71, 68.9%) followed by DVH (n = 20, 19.4%). Other indications are in Table 1. The mean total duration of treatment delay was 81.67 ± 17.15 days. While delay due to the Avastin® ban was 67.47 ± 4.8 days. Nearly half of the eyes had not received any injections from the time of their diagnosis (n = 46, 44.7%) while others had received one (n = 43, 41.7%) or two injections (n = 14, 13.6%) before.

**Table 1:** Mean central macular thickness in microns (CMT) with each diagnosis and its p-value CMT: central macular thickness; DME: diabetic macular edema; CRVO: central retinal vein occlusion; BRVO: branch retinal vein occlusion; AMD: age-related macular degeneration

Diagnosis	Mean CMT before (μm)	Mean CMT after (μm)	p-value
DME n = 71	354.2 ± 101.8	381.5 ± 102.2	<0.01
CRVO n = 5	510.8 ± 223.8	594.4 ± 265.5	0.07
BRVO n = 4	354.7 ± 49.8	397.5 ± 68.7	<0.05
Wet AMD n = 3	326 ± 38.5	470 ± 52.4	<0.05

The mean CMT before treatment delay was 362.7 ± 113.4 μm while after treatment delay was 398.38 ± 124 μm (p < 0.05). Among the 20 patients scheduled for IVB for DVH, 14 showed marked improvement (n = 14, 70%), while five showed no change in severity (n = 5, 20%), and only one (n = 1, 5%) had further worsening. Only three of these patients had received prior IVB. No significance was found between the resolution of VH with prior injection (p = 0.08).

**Figure 1:**
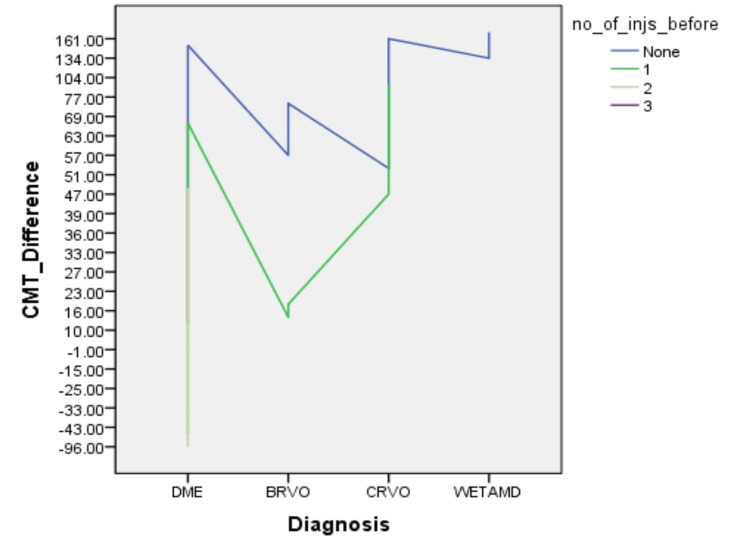
The correlation between the CMT Difference (before and after the treatment delay) and the number of injections for each diagnosis no_of_injs_before: number of injections given before; CMT: central macular thickness; DME: diabetic macular edema; CRVO: central retinal vein occlusion; BRVO: branch retinal vein occlusion; AMD: age-related macular degeneration

CMT difference was strongly correlated with the total duration of treatment delay (p < 0.01) and with the number of injections (p < 0.01) while no correlation was established between CMT difference and gender (p = 0.2). The difference in CMT was statistically significant for eyes that had not received any injections (p < 0.01) and for those that had received at least one injection (p < 0.01) before the treatment delay. However, the difference was not statistically significant (p = 0.1) for eyes that had received at least two injections before the treatment delay. Figure 1 shows the CMT difference for each diagnosis with the number of injections. The maximum CMT difference in our data is for DME and most of the eyes had not received any injection before the unprecedented treatment delay.

## Discussion

The local ban on intravitreal Avastin® injection was instated following an outbreak of IE, impacting a minimum of 68 eyes. Consequently, our ability to provide this routinely performed procedure was suspended for over eight weeks. In our study, we analyzed the impact of the Avastin® ban by comparing CMT on the OCT thickness map before and after the treatment delay and found a significant increase (p < 0.05).

Among the identified 377 patients for our study, only 23 (6.1%) were able to afford the alternate anti-VEGF IVI (Patizra/Eylea) privately during the Avastin® ban period reflect the deteriorating socioeconomic status of our population, and loopholes in healthcare access. Our results show the most common indication of IVB in our area is DME (n = 71, 68.9%) followed by DVH (n = 20, 19.4%) highlighting the burden of diabetes (26.3% prevalence by the second National Diabetes Survey of Pakistan (NDSP), 2016-2017) [11] and its related ocular complications due to poor control. Wet AMD being the last in line in the diagnosis shows the rarity of the disease in our part of the world compared to the West (1.56% prevalence in Pakistan [12] compared to 5.6% in the USA [13]).

We report a worsened CMT with all our indications including DME, RVO, and wet AMD with a delay of more than 11 weeks (81.67 ± 17.15). In literature, a delay of more than eight weeks has been described as adversely affecting the baseline CMT along with the VA in studies conducted during the COVID-lockdown period [14,15]. In our data, delay was most significant for our DME patients while in literature it has been wet AMD to be most adversely affected by a delay of treatment [15]. It can be attributed to the larger sample of DME in our data. Another contributing factor can be poorer diabetic control in our patients which is one of the known worse prognostic factors [16,17] primarily due to hurdles in health care access and disease awareness. Worsened CMT in other indications including RVO and wet AMD has been well documented before [15]. For RVO, our findings were similar to a previous study [18] indicating worsened effects of delayed treatment in CRVO than BRVO. In literature, the relationship between CMT and VA is emphasized [19]. However, our ability to assess VA was hindered by incomplete data in our system. The deterioration of CMT resulting from interrupted treatment is a clear indication of declining VA among our patients. Furthermore, in accordance with protocol I, reducing the edema in DME in the first year is of utmost importance as a transition from acute to chronicity of edema carries adverse anatomical and visual prognosis [20] indicating an increased burden of chronic DME in our patients.

Duration of action of IVB is claimed to be around eight weeks after which its effect is lost with progressive worsening of CMT with discontinuation of injection in a study conducted specifically for RVO [21]. In another study, IVB was found to be slow to act but long to clear up from vitreous in eyes with wet AMD (101.8 days ± 16.6) [22]. However, the data on exact pharmacokinetics is limited with Bevacizumab concentration of 1 g/ml at day 51 from the day of injection from the aqueous sample with some variability due to inevitable drug reflux from injection site and difference in vitreous volume in various eyes in a study before [23]. There is an absence of data in the literature on the duration of action of IVB in DR eyes. However, Protocol T has firmly established that VA at 12 weeks after receiving three consecutive monthly injections is positively correlated with vision outcomes over two years [24]. This underscores the significance of starting with regular monthly injections initially. A significant inverse relation of worsening CMT with the number of injected IVI was shown in our data with no prior injection showing maximum deterioration of CMT while eyes who had received at least two injections before the treatment interruption showed either improvement or stability of their CMT (p = 0.1). This suggests that eyes that have not received any treatment since diagnosis are at a risk of long-term deterioration in VA.

Where other indications showed worsening signs, DVH showed improvement in most of the patients (n = 14, 70%) only two of whom had received a single dose of IVB implying that observation alone might be sufficient for the hemorrhage to resolve. Although the efficacy of IVB in earlier resolution of DVH has been claimed by studies before [25,26] a larger study is still required to prove its long-term advantage. In our area where healthcare resources are already limited, more attention should be directed toward indications where an obvious benefit has been well documented in literature.

Similar to any medical procedure, IVIs are also associated with certain complications, the most concerning of which is IE because of its association with potential blindness [2]. However, its reported risk is very low (0.019%) [3]. Risk factors for post-IVI IE can be related to the drug (counterfeit drug, improper storage, lapse of cold chain), injection (contaminated needle, multiple use of same vial, improper sterilization technique), or patient (local or systemic infection, DM) [27]. The risk of IE with Bevacizumab (Avastin®) is slightly higher (3.64%) compared to other anti-VEGF injections (Ranibizumab 1.39%, Aflibercept 0.76%) [28], and one of the many reasons is the procurement of vials from unauthenticated sources and fake drug dealers. Avastin® is an anti-cancer drug manufactured by the Swiss pharmaceutical company Roche and is delivered to hospital pharmacies by local distributors in a larger volume vial of 4 ml. For ophthalmic purposes, smaller ampules are typically prepared from this vial either by compounding pharmacies or skilled operating theater nurses. This additional step between the manufacturer and the physician introduces another potential risk of contamination with this injection [29]. That is why IE with Avastin® injection is usually encountered in clusters. The ban on the sale and distribution of Avastin® by DRAP after an outbreak of IE in a local city was indeed a necessary step. However, while such measures are crucial for ensuring patient safety and emphasizing the need for strict monitoring and adherence to safety protocols, prolonged cessation also poses risks to our patients. The delay in treatment heightened the severity of the disease in many, highlighting the delicate balance between safety precautions and timely access to essential medical interventions.

To navigate similar situations in the future, maintaining a delicate balance between benefiting the patient and avoiding harm, we propose several steps. Ethical triaging, as previously suggested [30], can be implemented. This involves prioritizing those in urgent need of injections, such as patients with DME, RVO, and wet AMD. Cases of VH due to diabetes or other causes can be deferred until the safety of the drug is well established. Ensuring access to affordable alternative treatments after a specific brand has been banned especially for vulnerable populations with limited resources so that essential treatments remain accessible to all. To maintain up-to-date safety regulations in every set-up, regular visits by the drug authority team should be conducted. There should be a strict policy for reporting IE associated with any IVI by every setup to DRAP on a regular basis. In the event of an outbreak, local lockdowns should be implemented with thorough investigations. After ensuring safety, the drug should be made immediately accessible to those in need.

The data we have presented for one week provides a snapshot, but the true impact of the lockdown on patients receiving injections is likely far greater. The harm caused during this extended period may not be fully documented or captured, highlighting the need for comprehensive assessments and strategies to mitigate the impact of such prolonged disruptions on patient care.

## Conclusions

In conclusion, the prohibition of Avastin® for intraocular use reveals the challenges of balancing patient safety with the availability of crucial treatments. While safety is paramount, ensuring that effective and affordable alternatives are available is equally important. By improving compounding practices, enhancing regulatory oversight, promoting new treatment developments, and providing comprehensive patient support, the healthcare system can better navigate these challenges and ensure that patients receive the best possible care.
